# Effects of blood parasite infections on spatiotemporal migration patterns and activity budgets in a long‐distance migratory passerine

**DOI:** 10.1002/ece3.7030

**Published:** 2020-11-18

**Authors:** Tamara Emmenegger, Staffan Bensch, Steffen Hahn, Dmitry Kishkinev, Petr Procházka, Pavel Zehtindjiev, Silke Bauer

**Affiliations:** ^1^ Department of Bird Migration Swiss Ornithological Institute Sempach Switzerland; ^2^ Institute of Integrative Biology ETH Zurich Zürich Switzerland; ^3^ Molecular Ecology and Evolution Lab Department of Biology Lund University Lund Sweden; ^4^ School of Natural Sciences Bangor University Bangor UK; ^5^ Biological Station Rybachy Zoological Institute of Russian Academy of Sciences Rybachy Russia; ^6^ School of Life Sciences Keele University Keele UK; ^7^ Institute of Vertebrate Biology The Czech Academy of Sciences Brno Czech Republic; ^8^ Institute of Biodiversity and Ecosystem Research Bulgarian Academy of Sciences Sofia Bulgaria

**Keywords:** activity, biologging, bird migration, flight height, great reed warbler, *Haemoproteus*, migration timing, parasites, *Plasmodium*, resting

## Abstract

How blood parasite infections influence the migration of hosts remains a lively debated issue as past studies found negative, positive, or no response to infections. This particularly applies to small birds, for which monitoring of detailed migration behavior over a whole annual cycle has been technically unachievable so far. Here, we investigate how bird migration is influenced by parasite infections. To this end, we tracked great reed warblers (*Acrocephalus arundinaceus*) with multisensor loggers, characterized general migration patterns as well as detailed flight bout durations, resting times and flight heights, and related these to the genus and intensity of their avian haemosporidian infections. We found migration distances to be shorter and the onset of autumn migration to be delayed with increasing intensity of blood parasite infection, in particular for birds with *Plasmodium* and mixed‐genus infections. Additionally, the durations of migratory flight bout were prolonged for infected compared to uninfected birds. But since severely infected birds and particularly birds with mixed‐genus infections had shorter resting times, initial delays seemed to be compensated for and the timing in other periods of the annual cycle was not compromised by infection. Overall, our multisensor logger approach revealed that avian blood parasites have mostly subtle effects on migratory performance and that effects can occur in specific periods of the year only.

## Graphical abstract

Using multisensor loggers and real‐time quantitative PCR, we found that infections with avian haemosporidian parasites have diverse, but relatively weak effects on the migration of great reed warblers. Our findings indicate that some parasite effects can be compensated and that avian hosts can cope with a broad range of chronic infection intensities. This implies that infected hosts maintain their migratory capacity and thus also their potential for spreading parasites on their cross‐continental journeys.

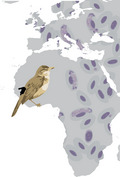

## 1 INTRODUCTION

Infection‐related deviations in host migration performance have already been described for a variety of parasite and host taxa: Monarch butterflies migrated slower when infected by ectoparasites (Bradley & Altizer, 2005), reindeer herds with more ectoparasites performed shorter migrations (Folstad et al., 1991), and the timing of arrival at the breeding site was delayed for blood parasite infected compared to uninfected passerines (Rätti et al., 1993). A recent meta‐analysis suggested movement capacity and phenology of migratory animals to be moderately affected by the status and intensity of infections, while body condition and survival were only weakly associated with infection (Risely et al., 2018). Nevertheless, the magnitude of parasite effects on migratory hosts and, particularly, how effects depend on the taxon and severity of infection, remain unresolved.

In migratory birds, parasites might affect host migration via several mechanistic pathways: Parasites could (a) directly reduce physiological performance and thereby lead to slower flight. Early seminal works indicated that *Plasmodium* infections decreased the oxygen consumption rates of experimentally infected canaries during nocturnal rest (Hayworth et al., 1987) and naturally infected lizards during exercise (Schall et al., 1982). In contrast, the metabolic rates of a migratory passerine were not significantly influenced by infection neither during resting nor during exercise both for natural haemosporidian infections and experimental *Plasmodium* infections (Hahn et al., 2018).

Alternatively, parasites could (b) indirectly impair host migration, for example, by slowing down fueling, prolonging stopovers or increasing the need for resting after endurance flights. For instance, benign avian influenza infections lowered the feeding rates and prolonged stopover durations in migratory swans (van Gils et al., 2007) and infections with multiple intestinal parasites were correlated with delayed spring migration timing in passerines (López et al., 2013).

Yet, regardless of the mechanisms behind parasite effects on host migration, such changes are thought to cascade through to breeding, with delayed arrivals and late onset of breeding lowering individual fitness (Kokko, 1999). However, so far, the insight into infection‐related changes of host migration had been restricted to (a) basic migration parameters measurable by standard methods (e.g., geolocation, telemetry, and satellite tracking) and (b) snapshots of certain periods of the annual cycle. A full‐migration approach relating detailed migration behavior to individual infection parameters is still missing for wild birds.

Therefore, we used multisensor loggers to describe detailed migration patterns of great reed warblers (*Acrocephalus arundinaceus*) and related these patterns to the hosts’ blood parasite infections (genera *Plasmodium* and *Haemoproteus* within Haemosporida). Multisensor loggers do not only enable geolocation by recording light, but also allow compiling individual activity and behavior by recording accelero‐ and barometric data (Liechti et al., 2018). The recorded patterns were then related to the parasite genus and the individual intensities of avian haemosporidian parasite infections assessed by real‐time quantitative PCR. Specifically, we related these infection parameters measured at the deployment and retrieval of the loggers to several key migration traits: distance, duration, and speed of migration, the timing of autumn and spring migration, the duration of migratory flight bouts and resting periods, and flight height.

If infections affect migration throughout on a broad scale, we expect infected individuals to migrate shorter distances or to take longer for the same distance. If migration distance would vary for infections with different parasite genera, this could both signify a parasite effect on migration performance and a differential probability of getting infected with different parasites in various regions of the nonbreeding range. Depending on how infections affects migration timing, we may expect the following outcomes: If blood parasites hamper the preparation for migration, we expect that infected individuals to depart with delays. If, in contrast, blood parasites affect the progression of migration, we rather expect arrivals to be delayed. By using multisensor loggers, we also expect insights into the mechanisms behind parasite on migration patterns on a finer scale: If blood parasites impair flight, we expect infected birds to fly slower and their migratory flight bouts to be shorter (if fuel is limiting) or longer (if infected birds compensate for slower flight). If, in turn, blood parasites impair fueling rates or increase energy expenditure, infected individuals are expected to need longer resting times. Finally, if partial oxygen pressures experienced aloft are limiting for migrating birds, we expect infected individuals to fly at lower altitudes. All these potential effects are expected to increase with growing intensity of infection and are to some degree expected to differ between parasite genera, as *Haemoproteus* and *Plasmodium* infections are known to differ in their average pathogenicity and co‐infections with several genera are known to be most virulent (Valkiūnas, 2005).

## 2 MATERIAL AND METHODS

### 2.1 Study species and field sites

**Figure 1:**
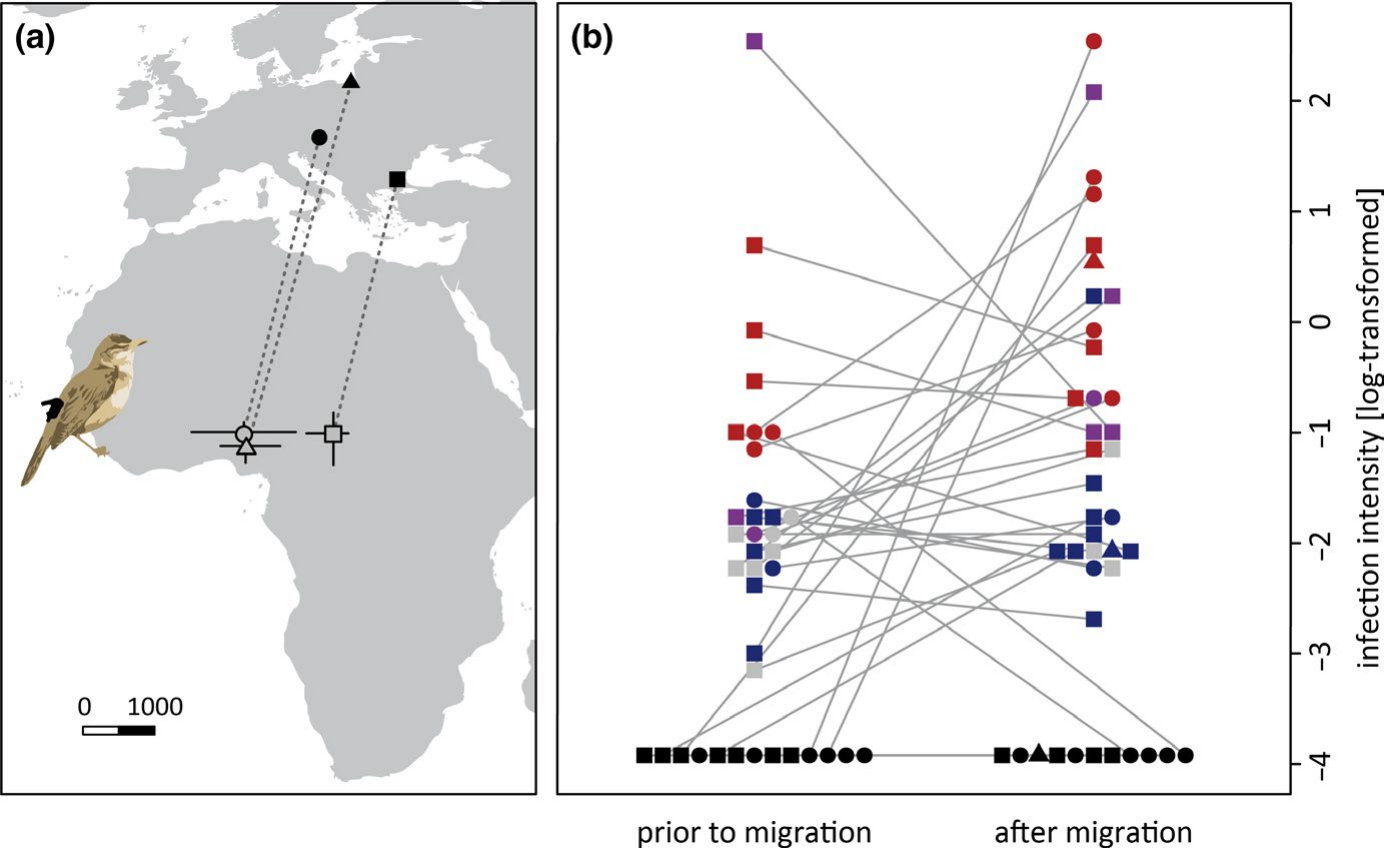
The breeding sites and site‐specific nonbreeding ranges of the tracked great reed warblers (a) as well as the individual genus and intensity of haemosporidian infections prior to and after the tracked migrations (b). Birds breeding in Bulgaria (closed square), Czech Republic (closed circle), and Russia (closed triangle) spent the nonbreeding period in sub‐Saharan Africa (open symbols = site‐specific median, ±25%–75% quantiles of the individual nonbreeding sites). The haemosporidian infections were generally more intense after than prior to the tracked migrations, and*Plasmodium*infections (blue) tended to be less intense than*Haemoproteus*infections (red) and mixed‐genus infections (purple). Note that for some samples parasite genus is unknown (gray) and some points are unconnected as the pre‐migration blood sample was missing (*n* = 3)

We investigated great reed warblers (*A. arundinaceus*) breeding at three study sites in Bulgaria (BG, Kalimok Biological Station, 44.00°N 26.45°E), in the Czech Republic (CZ, Mutěnice, 48.90°N, 17.05°E), and in western Russia (RU, Rybachy, 55.15°N, 20.85°E). Great reed warblers breed in reed beds along fresh or brackish standing water and spend the nonbreeding season in sub‐Saharan Africa (Koleček et al., 2016 and Figure 1a). They often harbor haemosporidian parasites—a widespread group of blood parasites which are transmitted by dipteran vectors—and within the two genera *Plasmodium* and *Haemoproteus* 29 genetic lineages have been recorded for great reed warblers (MalAvi database; Bensch et al., 2009; accessed on the 13.06.2019). The two parasite genera and their lineages can greatly vary in their virulence. While many infections are benign, some infected hosts can show fatigue and lose appetite during the acute phase or rarely even die (Lapointe et al., 2012). Most avian blood parasites infections become chronic and the minor effects of chronic infections are known to accumulate, for example, resulting in reduced lifetime reproductive success in great reed warblers (Asghar et al., 2011).

### 2.2 Study design and data acquisition

In spring and summer 2015–2017, we captured great reed warblers with mist nets, and all 351 adults were sexed, aged, weighed, and ringed. Approximately three quarters of these birds (*n*_TOTAL_ = 255; *n*_BG.2015_ = 70, *n*_BG.2016_ = 80, *n*_CZ.2016_ = 40, *n*_CZ.2017_ = 30, *n*_RU.2016_ = 35) were equipped with multisensor loggers (SOI‐GDL3pam)—devices that record light intensity, activity and atmospheric pressure (Liechti et al., 2018). The loggers weighed approximately 1.4 g, which amounts to 4.5% of the mean body mass of our study species. The remaining birds (*n*_TOTAL_ = 96; *n*_BG.2015_ = 38, *n*_BG.2016_ = 40, *n*_CZ.2016_ = 12, *n*_CZ.2017_ = 6) served as a procedural control group (in BG and CZ only). The number of birds captured in RU was too low for establishing a control group. In 2016 –2018, we recaptured 40 of the logger birds (*n*_BG.2016_ = 13, *n*_BG.2017_ = 10, *n*_CZ.2017_ = 5, *n*_CZ.2018_ = 9, *n*_RU.2017_ = 3) and 29 control birds (*n*_BG.2016_ = 17, *n*_BG.2017_ = 7, *n*_CZ.2017_ = 5, *n*_CZ.2018_ = 0). Thus, the recapture rates were lower in the logger birds compared to the control birds (15.7% for logged vs. 30.2% for control birds), which indicates a lower apparent survival that may either be caused by higher mortality or lower side fidelity in logger birds (Arlt et al., 2013). Such logger effects should be considered when interpreting absolute measures of host migration characteristics. Comparing only logged birds between each other, the comparative part of our analysis should not be biased by potential logger effects.

### 2.3 Geolocation

To calculate migration distances, we first determined the main nonbreeding sites from light‐level data, using the package GeoLight (Lisovski & Hahn, 2012) following Koleček et al. (2018). In brief, sunrise and sunset times were determined by the function twilightCalc, setting an arbitrary light‐level threshold. The stationary periods were delimited with the function changeLight, setting the quantile and days parameters to 0.9 and 3, respectively. The sun events from the first postbreeding period were used to calibrate the data using the function getElevation. Finally, the sun elevation angle determined by the calibration was fed to the coord function for calculating raw positions. These positions were aggregated to main nonbreeding stationary sites (i.e., mode of the longitudes and latitudes of all raw positions within each main nonbreeding stationary period). As great reed warblers use one or two distinct main nonbreeding sites (Koleček et al., 2018), the autumn migration distance was calculated as the loxodromic distance between the breeding site and the first nonbreeding site and spring migration distance as the distance from the second nonbreeding site back to the breeding site. For birds with only one nonbreeding site, the two migration distances were identical.

As the light sensor of one logging device failed, this bird was excluded from all analyses which involved migration distance but retained for all other models. When devices stopped recording data within the nonbreeding period (*n* = 9), we only analyzed autumn migration (see Table S1).

### 2.4 Activity analysis

The multisensor loggers measured light intensity and acceleration every 5 min and air pressure and ambient air temperature every 30 min. Acceleration was sensed along the *z*‐axis (i.e., the bird's dorsoventral axis) with a frequency of 10 Hz for 3.2‐s period. The mean of these 32 values was stored as “pitch” (corresponding to the relative position of the body axis) and the sum of the 31 differences between these values as “activity” (corresponding to the movements deviating from the mean body axis). Following Liechti et al. (2018), we used the bimodal frequency distribution of individual activity values to assign them to three behavioral categories: “resting” when activity levels were zero, “active” when activity levels were above zero but below the local minimum in the frequency distribution and “flight” when activity levels exceeded the local minimum in the frequency distribution. For “migratory flight bout,” activity had to be continuously categorized as “flight” for at least 1 hr. After excluding flight bouts during the intratropical migration period, the remaining migratory flight bouts were assigned to the two main migration periods in autumn and spring and used to delimit the timing of migration events: The date/time when the first flight bout started was defined as “departure” and the date/time when the last flight bout ended as “arrival.” Consequently, “resting times” were the periods in between migratory flight bouts. The average migration speed was calculated by dividing the total migration duration (time from departure to arrival in days) by the total migration distance (in km) for autumn and spring separately. Finally, we extracted the duration for each flight bout and applied a hypsometric function to estimate flight height based on the air pressure measured on the birds (Stull, 2017).

Six birds were excluded from the analysis of activity‐related variables on spring migration because their devices stopped recording acceleration en route (see Table S1).

### 2.5 Determination of infection intensity and parasite genus

We collected blood samples for 36 birds (*n*_BG_ = 22, *n*_CZ_ = 14) prior to the tracked migration and 39 birds (*n*_BG_ = 22, *n*_CZ_ = 14, *n*_RU_ = 3) after retrieval of the loggers the following spring. We punctured the vena brachialis and the initial blood drop was collected to prepare thin blood smears, which were air‐dried, fixed in absolute MetOH, and dyed with Giemsa stain. For 35 of the 36 first and all 39 s samplings, the remaining blood was stored in SET buffer or absolute EtOH for later molecular analysis. From these samples, we extracted DNA (DNeasy blood and tissue kit, Qiagen), diluted it to 1 ng/µl, and performed real‐time quantitative PCRs (qPCR; using Sybr Green protocol; Invitrogen™ and Mx3000P qPCR system; Stratagene). We used general primers targeting a short (153 bp) region of the RNA subunits in the mitochondrial genome of haemosporidians (RNA_343F = 5′ GCT CAC GCA TCG CTT CT 3′, RNA_496R = 5′ GAC CGG TCA TTT TCT TTG 3′; Fallon et al., 2003). These primers jointly amplify infections by parasites of the genera *Plasmodium* and *Haemoproteus*. For each qPCR, we used positive template dilution series and negative template controls along with the samples to be quantified in dupli‐ or triplicates. We reran samples with unclear dissociation curves or amplification plots twice. The slopes in the standard curves of our final qPCRs ranged between −2.6 and −3.9 indicating appropriate reaction efficiency. To adjust the parasite quantification for differences in total DNA concentrations, we used vertebrate‐specific primers for a nuclear (single copy) noncoding region (SFSR_3Fb = 5′ ACT AGC CCT TTC AGC GTC ATG T 3′, SFSR_3Rb = 5′ CAT GCT CGG GAA CCA AAG G 3′; Asghar et al., 2011) for samples which turned out to be infected in the qPCR with the parasite primers. Thereof, we calculated relative parasitaemia by standardizing the quantity yielded from the parasite‐specific qPCR with total DNA concentration determined by the vertebrate‐specific qPCR.

It has previously been shown that this procedure yields infection intensity estimates, which agree well with parasitaemia determined by microscopy for *Haemoproteus* parasites of the lineage GRW1 (Asghar et al., 2011). However, when interpreting qPCR‐derived intensities of natural infections, one should consider that (a) blood parasites from different genera can differ in their typical infection intensities, as relatively mild *Haemoproteus* infections might have similar qPCR intensities like relatively high‐intensity *Plasmodium* infections and that (b) relative intensities assessed by qPCR disparately correspond to intensities determined by classical microscopy, because some developmental stages, for example, *Plasmodium* meronts contain numerous copies of DNA per infected erythrocyte, which would increase the relative intensity measured by qPCR compared to parasitaemia measured by microscopy.

If no blood was stored for molecular analysis, the respective samples were excluded from analyses involving infection intensity (*n* = 6). For one of these individuals, we found no haemosporidian parasites when screening 100 fields with 1,000× magnification under a light microscope and set the infection intensity to zero.

To retrospectively assign the parasite genus for infected samples, we used the results of qPCRs done with genus‐specific primer pairs (GRW1/8F + GRW1/9R for *Haemoproteus* as well as GRW2/8F + GRW2/9R and GRW4/11F + GRW4/11RCAT for *Plasmodium*; Asghar et al. (2011)) where available, or sequenced the amplicons of a standard nested PCR (primer pairs HaemNF1 + HaemNR3 and HaemF + HaemR2; Hellgren et al. (2004)). For eight samples the results of the genus‐specific qPCRs were inconclusive and for two samples the chromatograms showed mixed template patterns to a degree that genus could not be assigned, resulting in ten missing values for parasite genus. All other samples could be assigned to *Plasmodium*, *Haemoproteus*, mixed‐genus infection or no genus, if the infection status was “uninfected.”

### 2.6 Statistics

We built models that relate infection parameters at deployment and retrieval of the loggers to variables related to host migration in autumn and spring, respectively. To this end, we log‐transformed the relative infection intensities (function logst; see Stahel, 2017) and centered all performance‐related variables within sites by subtracting the site‐specific mean, to avoid breeding origin of the birds as a confounding factor.

We built three series of models, which include either the intensity of infection, the parasite genus or both as explanatory variables. Using only infection intensity allowed the detection of general intensity‐related parasite effects (as, e.g., caused by a reduction of the oxygen transport capacity of parasitized erythrocytes) and avoided reduction of sample size due to missing parasite genus data. Using parasite genus only enabled detection of parasite effects independent from the intensity of infection (e.g., due to systemic effects of certain stages in the life cycle of a specific parasite genus). And the combination of both variables allowed detection of effects of one variable when controlling for the other and therefore enables further interpretation of interrelated effects. If not stated differently, estimates presented in the results section originate from models with both focal explanatory variables. In all models, we used sex and, if applicable, season to control for potential confounding effects. For season‐specific response variables, that is, the timing of migration events, we built linear models (LM; function lm of the basic R package stats), and for all other response variables, we built linear mixed‐effect models (LME; function lmer of the R package lme4, Bates et al., 2015) with individual as a random factor (see Table S2 for a variable glossary and Table S3 for details of model design).

For both LMs and LMEs, we applied Bayesian simulation techniques to compute posterior distributions of model parameters (function sim of the R package arm, Gelman & Su, 2016) and considered estimates of fixed effects which did not overlap with zero to be significant on an alpha level of 5%. All calculations and models were run in the R environment (R Core Team, 2019).

## 3 RESULTS

### 3.1 Parasite infections

Overall, approximately 70% of the great reed warblers harbored blood parasite infections (24 of 37 individuals prior to migration and 29 of 40 after migration). These infections comprised seven and eleven *Plasmodium*, seven and ten *Haemoproteus* as well as three and five mixed‐genus infections prior to and after migration, respectively (Figure 1b). The relative infection intensities assessed by qPCR were slightly higher after (median: 0.10, 25%–75% quantiles: 0.08–1.82) compared to before migration (0.02, 0.008–0.10; see Figure 1b).

### 3.2 Host migration characteristics

Basic migration measures. The nonbreeding sites of tracked individuals roughly reflected the longitudinal pattern of the three study sites, with larger overlaps toward the West of the nonbreeding range (Figure 1a). The migration distances were shorter in autumn (4,207 km; 2,940–5,900 km) as compared to spring (4,417 km; 3,240–5,770 km). To cover these distances, the birds travelled on average 43 days (11–103 days) in autumn and 35 days (8–96 days) in spring. None of the three basic migration measures differed between the sexes and the tendency for advanced migration of males was not statistically significant.

#### 3.2.1 Flight bouts

Birds completed autumn migration with about 13 flight bouts (median; range: 7–27) and spring migration with 14 (7–32). On average, migratory flight bouts had a length of 7 hr, but with extreme nonstop flights lasting up to 44 hr. The total flight duration used for covering the full‐migration distance was 88 hr (57–153 hr) in autumn and 106 hr (73–242 hr) in spring.

#### 3.2.2 Resting times

Between migratory flight bouts, the birds rested on average for 18 hr in autumn and 16 hr in spring (median of the individual durations). Maximum individual resting times ranged between 62 and 928 hr in autumn and between 19 and 742 hr in spring.

#### 3.2.3 Flight height

Mean individual flight height was estimated at 1,055 m a.s.l. (range: 593–1,701 m a.s.l.) in autumn and 1,512 m a.s.l. (759–2,377 m a.s.l.) in spring, but maximum individual flight heights reached up to 4,396 and 4,850 m a.s.l. during autumn and spring migration, respectively.

### 3.3 Relationships between parasite infection and host migration

**Figure 2:**
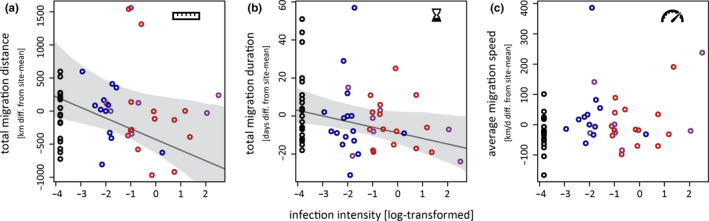
Effects of parasite infection on basic parameters of great reed warblers’ migration. While the total migration distance (a) and the total migration duration (b) of infected great reed warblers were significantly related to the intensity, but not the genus (open symbols:*Plasmodium* = blue,*Haemoproteus* = red, mixed‐genus = purple) of their haemosporidian infections, the resulting average migration speed (c) was statistically unrelated to both infection parameters

Individuals with higher intensity of infection migrated significantly less far (*β* = −161.38, CrI = −314.27 to −11.05; Figure 2a) and within shorter time (*β* = −2.77, CrI = −5.57 to −0.27; model with intensity as the only focal explanatory variable; Figure 2b) than hosts with lower infection intensities. The resulting average migration speed was not significantly related to the intensity of infection (*β* = 19.83, CrI = −8.80 to 48.39; Figure 2c). Parasite genus was not related to any of the three basic migration parameters (see Table S3 for model estimates).

**Figure 3:**
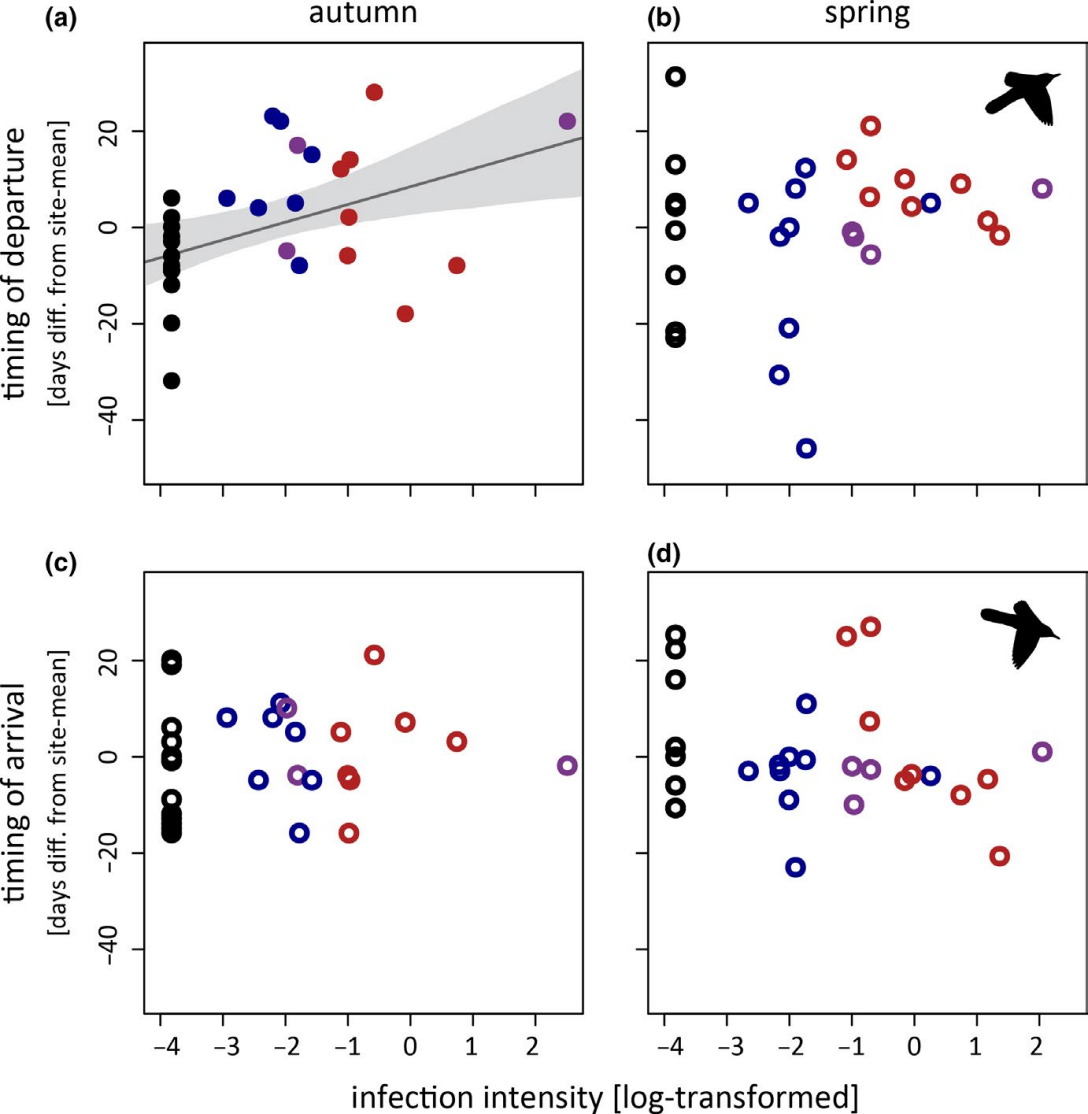
The timing of departures (a + c) and arrivals (b + d) of great reed warblers in autumn (a + b) and spring (c + d) as dependent on the haemosporidian infection. Only the departure for autumn migration was significantly related to genus (LM fit ± CrI; closed symbols:*Plasmodium* = blue,*Haemoproteus* = red, mixed‐genus = purple) and intensity of infections. All other timing events were statistically unrelated to infection parameters (open symbols)

With increasing infection intensities, birds departed progressively later for autumn migration (*β* = 3.64, CrI = 1.15–6.16; model with intensity as the only focal explanatory variable; Figure 3a). This pattern was also reflected in the model with parasite genus as the only focal explanatory variable: Birds infected by *Plasmodium* (*β* = 16.77, CrI = 5.73–27.44) or both genera (*β* = 19.27, CrI = 4.16–34.22) departed significantly later than uninfected birds (but not significant for *Haemoproteus:* *β* = 10.05, CrI = −1.11–21.53). The timing of all other migration events was neither related to parasite genus nor to infection intensity (see Figure 3b–d and Table S3 for model estimates).

**Figure 4:**
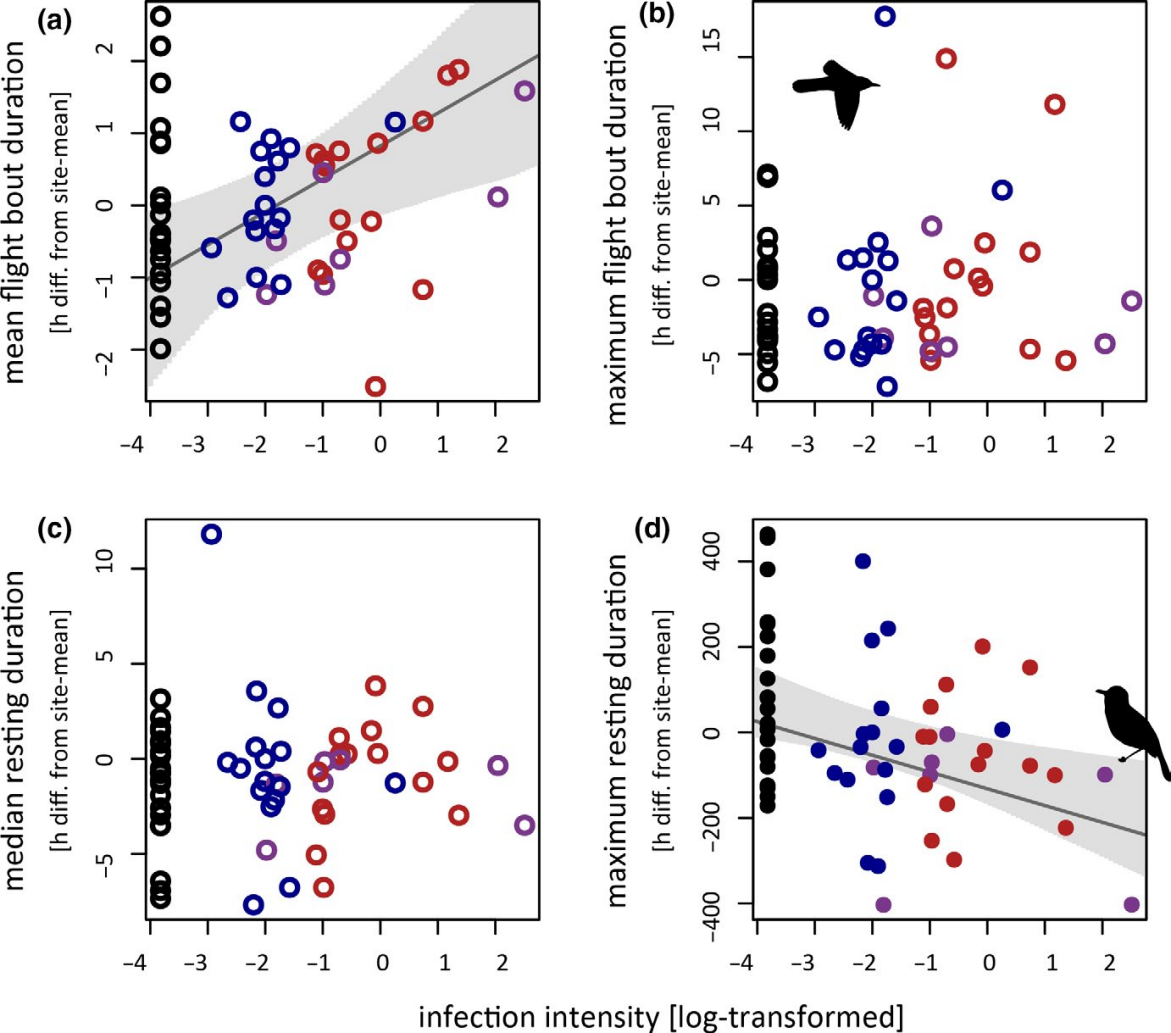
The interrelations between the haemosporidian infections of tagged great reed warbler with the mean (a) and the maximum (b) of individual flight bout durations as well as the median (c) and the maximum (d) individual resting durations. While mean flight times were merely related to infection intensity (LME fit ± CrI; open symbols:*Plasmodium* = blue,*Haemoproteus* = red, mixed‐genus = purple), the maximal resting times were significantly related to both the intensity and the genus of the hosts’ haemosporidian infections (LME fit ± CrI; closed symbols)

The individual mean of flight bout durations was positively related to intensity of infections (*β* = 0.46, CrI = 0.11–0.81; Figure 4a), but not to parasite genus. The maximal flight bout durations were neither statistically related to parasite genus nor to infection intensity (see Figure 4b and Table S3 for model estimates).

The maximum of the individual resting durations was related to infection intensity (*β* = −43.45, CrI = −80.96 to −6.46; model with infection intensity as the only focal explanatory variable) and parasite genus (*Plasmodium*: *β* = −118.92, CrI = −243.97 to 0.68; *Haemoproteus*: *β* = −116.58, CrI = −246.53 to 11.44; mixed‐genus: *β* = −268.92, CrI = −434.05 to −103.35; all compared to uninfected birds in the model with parasite genus as the only focal explanatory variable) with significantly shorter maximum resting durations for birds with higher infection intensity of infection as well as birds with mixed‐genus infections (Figure 4c). In contrast, the individual median resting durations were neither significantly related to parasite genus nor to infection intensity (see Figure 4d and Table S3 for model estimates). The exclusion of an outlying median resting duration value of about 90 hr (more than 60 hr more than the site‐specific average) from a bird with an unusual migration strategy (i.e., only few and mostly nonconsecutive flight nights in autumn) did not change this result.

While mean individual flight heights were positively related to infection intensity (*β* = 33.21, CrI = −27.50 to 96.39; model with intensity as the only focal explanatory variable), maximum flight heights differed between parasite genera, with *Plasmodium* infected birds flying significantly lower (*β* = −1,142.93, CrI = −1,977.13 to −325.91) than uninfected birds (but not significant for *Haemoproteus*: *β* = −1,117.70, CrI = −2,383.22 to 122.00 and mixed‐genus: *β* = −1,250.35, CrI = −2,573.68 to 82.22).

## 4 DISCUSSION

### 4.1 Parasite infections and basic migration patterns

Our findings suggest that great reed warblers with more severe haemosporidian infections migrate shorter distances and take less time for the whole migration than birds with mild or no infections, resulting in similar average migration speeds among all tracked individuals. However, compared to total migration distances (on average 4,207 km in autumn and 4,417 km in spring) the estimated difference of 82 km (CrI = 33–144 km) between noninfected individuals and individuals with an average infection intensity for our study is quite small.

In general, such a relation between infection and migration distance can be interpreted both as a parasite effect on host migration or as an effect of host migration patterns on the resulting parasite infections. The former implicates that infections reduce the hosts’ propensity or ability to migrate which ultimately results in shorter migration distances, similar to what has been found for swans with low‐pathogenic influenza infections (van Gils et al., 2007). The latter applies if the region and habitats where the birds spend the nonbreeding season critically co‐determine which parasite infections birds acquire. Indeed, birds using different molting sites also differed in the probability to get infected with haemosporidian parasites (Yohannes et al., 2008). As region‐specific infection pressures would probably also influence the composition of the parasite fauna harbored, we would also expect a relation between migration distance and parasite genus—a prediction that our study does not confirm. Even if our study cannot disentangle the two potential explanations for the relation between infection and migration distance, the direct link between migration distance and infection intensity and the lack of a genus effect indicate a parasite effect on migration performance.

### 4.2 Parasite infections and migration timing

Among the four timing events investigated, only the onset of autumn migration was significantly delayed—with noninfected individuals departing on average 6 days earlier (CrI = 4.6–7.8 days) than individuals with an average infection intensity within our study. However, this pattern was strongly influenced by one individual with a particularly high infection intensity and late departure.

In general, infections could delay migration via two nonexclusive mechanistic pathways. First, parasite infections might affect preparations for migration, for example, by reducing fueling rates (van Gils et al., 2007). However, if this applies, we would also expect a delayed spring departure, particularly because infections are known to be relapsing in spring (Valkiūnas et al., 2004) and (re‐)infections taking place during the nonbreeding period (Hasselquist et al., 2007). However, spring migration timing was not affected by infection intensity in our study. Alternatively, delays in the onset of autumn migration could be carried over from the breeding season, for example, owing to higher energetic demands during territory establishment and/or the chick raising phase. Such processes could have already hampered territory defense or parental care and subsequently delayed the onset or the progression of the preparation for migration—rather than parasites directly hampering preparation for migration.

As *Plasmodium* infections (known to occur with lower average infection intensities than *Haemoproteus* parasites) and infections with both haemosporidian genera in particular led to such delays indicates that it is not infection intensity per se but rather a systemic effect of certain stages in the life cycle of parasites belonging to the genus *Plasmodium*. Studies on differential pathogenicity of haemosporidians revealed that specific lineages of *Plasmodium* parasites can be much more pathogenic than *Haemoproteus* infections, despite their relatively low parasitaemia (Himmel et al., 2020; Ilgūnas et al., 2016). Unfortunately, our sample size is insufficient for a lineage‐specific comparison. It remains for future studies to quantify how specific the different genera and lineages affect their hosts during breeding and how these effects carry over into the migration period. Future studies should also aim at better covering the possible spectrum of infection intensities more equally, for example, by controlled infection experiments or at disentangling the proximate causes for delayed initiation of autumn migration, for example, by experimentally manipulating clutch sizes or levels of parasitaemia in breeding adults and tracking their departure dates using telemetry.

As indicated above, our data did not confirm infection‐induced delays on spring migration as found in earlier studies (Emmenegger et al., 2018; López et al., 2013). It seems that the generally mild effects of blood parasites on host migration timing are rather species‐specific and might be compensated by great reed warblers (e.g., by adjusting resting times, see next section).

### 4.3 Parasite infections and detailed migration behavior

Compared to classic light‐level geolocators that only approximate departures and arrivals (Lisovski et al., 2012), multisensor tracking devices allow estimating flight heights and clearly delimiting flight from resting periods (Liechti et al., 2018). Owing to this additional information, we could reveal that increased infection intensities are associated with higher mean flight altitudes, longer mean flight bout durations, and shorter maximum resting durations.

Explaining our findings of higher mean flight altitudes for more heavily infected birds but lower maximum flight heights for individuals infected with certain parasite genera is not straightforward. But it rejects our initial hypothesis that the partial oxygen pressures on great reed warblers’ flight altitudes are limiting. However, this does not necessarily imply that higher infection intensities or more limiting oxygen concentrations could not compromise migration behavior of different bird species and/or on other flyways flight performance.

We also found mean, but not the maximum, flight bout durations were related to parasite infection, illustrating that small birds are capable to perform astonishing nonstop flights of up to 44 hr regardless of their infection status. Both maximal flight bout durations and maximal flight heights were reached during Sahara crossing, and according to the timing of these extreme flights, we assume that birds avoid unfavorable flight conditions and landing in hostile habitats, even forcing typical nocturnal migrant to occasionally flying into the day (also see exemplary Figure S1 and Adamík et al., 2016). The longer mean flight bout durations with increasing infection intensity indicate that more heavily infected individuals need more time for covering the same distances rather than that these longer flights translate into covering more distance (see section on spatial migration patterns above).

Additionally, we found the maximum, but not the mean, resting durations to be reduced by infection, suggesting that, regardless of infection status, migrating birds need a certain minimum recovery period between consecutive migratory flight bouts. But extensive stopover periods can be shortened to compensate for delays—may they be caused by infections or other factors. This also implies that focusing on a single migration variable (e.g., the arrival at a stopover or breeding site) may be insufficient, as parasite effects on timing in one phase of the annual cycle can be compensated with adjustments in other phases. Whether these adjustments come with the drawback of being more exposed to predators due to compensatory feeding activity or of continuing migration in worse condition as shorter resting times cannot be compensated by extended feeding, cannot be tested with our data set.

In conclusion, using multisensor loggers enabled to describe detailed patterns in individual migration behaviors and how infections with parasites affect these. We found that chronic infections with avian blood parasites have diverse, but relatively weak effects on the migration performance of great reed warblers. Our results also indicate that birds can compensate some effects of parasites arising in certain periods of the annual cycle, which thus might not be detectable anymore in subsequent periods. This indicates that hosts can cope with a broad range of chronic infection intensities, maintaining most of their migratory capacity and thus also their potential for spreading parasites (Bauer & Hoye, 2014).

## CONFLICT OF INTEREST

None declared.

## AUTHOR CONTRIBUTIONS

**Tamara Emmenegger:** Conceptualization (equal); Data curation (lead); Formal analysis (equal); Investigation (equal); Methodology (equal); Project administration (supporting); Visualization (lead); Writing‐original draft (lead); Writing‐review & editing (lead). **Staffan Bensch:** Investigation (equal); Methodology (equal); Project administration (supporting); Resources (supporting); Supervision (supporting); Writing‐review & editing (supporting). **Steffen Hahn:** Conceptualization (equal); Formal analysis (supporting); Funding acquisition (equal); Investigation (equal); Methodology (equal); Project administration (equal); Resources (equal); Supervision (supporting); Validation (equal); Writing‐original draft (supporting); Writing‐review & editing (supporting). **Dmitry Kishkinev:** Data curation (supporting); Funding acquisition (supporting); Investigation (equal); Project administration (supporting); Resources (supporting); Writing‐review & editing (supporting). **Petr Procházka:** Data curation (supporting); Funding acquisition (supporting); Investigation (equal); Project administration (supporting); Resources (supporting); Writing‐review & editing (supporting). **Pavel Zehtindjiev:** Data curation (supporting); Funding acquisition (supporting); Investigation (equal); Project administration (supporting); Resources (supporting); Writing‐review & editing (supporting). **Silke Bauer:** Conceptualization (equal); Formal analysis (equal); Funding acquisition (equal); Investigation (supporting); Methodology (equal); Project administration (equal); Resources (equal); Supervision (lead); Validation (equal); Visualization (supporting); Writing‐original draft (supporting); Writing‐review & editing (equal).

## Supporting information

Supplementary Material

## Data Availability

The datasets analyzed and generated for this study are available on Zenodo (raw logger data: http://doi.org/10.5281/zenodo.4017739; migration variables and infection data: http://doi.org/10.5281/zenodo.4022516).
