# Supplementary material for: Effects of blood parasite infections on spatiotemporal migration patterns and activity budgets in a long‐distance migratory passerine
Source: Ecol Evol. 2020 Nov 18;11(2):753–62. doi: 10.1002/ece3.7030 (PMC7820147; doi:10.1002/ece3.7030)
Supplement: Supplementary file 1 — Supplementary Material [file ECE3-11-753-s001.docx]

Appendix

Table S1: Overview on logger data and type of blood samples available from the tracked birds - sorted by logger ID.

| general information | | | | light data | | multi-sensor data | | blood smear | | blood in SET | |
| --- | --- | --- | --- | --- | --- | --- | --- | --- | --- | --- | --- |
| logger id | ring id | study site | deployment year | autumn | spring | autumn | spring | before migration | after migration | before migration | after migration |
| 14AZ | 1-545764 | bg | 2015 | 1 | 1 | 1 | 1 | 1 | 0 | 0 | 0 |
| 14ES | 1-545798 | bg | 2015 | 1 | 1 | 1 | 1 | 1 | 1 | 1 | 1 |
| 14FF | 1-545865 | bg | 2015 | 1 | 1 | 1 | 1 | 1 | 1 | 1 | 1 |
| 14GD | 1-545690 | bg | 2015 | 1 | 1 | 1 | 1 | 1 | 1 | 1 | 1 |
| 14GF | 1-545842 | bg | 2015 | 1 | 1 | 1 | 1 | 1 | 1 | 1 | 1 |
| 14GG | 1-545679 | bg | 2015 | 1 | 1 | 1 | 1 | 1 | 1 | 1 | 1 |
| 14GH | 1-545683 | bg | 2015 | 1 | 1 | 1 | 1 | 1 | 1 | 1 | 1 |
| 14GS | 1-545763 | bg | 2015 | 1 | 1 | 1 | 1 | 1 | 1 | 1 | 1 |
| 14GW | 1-545859 | bg | 2015 | 0 | 0 | 1 | 1 | 1 | 1 | 1 | 1 |
| 14HA | 1-545681 | bg | 2015 | 1 | 1 | 1 | 1 | 1 | 1 | 1 | 1 |
| 14HB | 1-545703 | bg | 2015 | 1 | 0 | 1 | 0 | 1 | 1 | 1 | 1 |
| 14HC | 1-545680 | bg | 2015 | 1 | 1 | 1 | 1 | 1 | 1 | 1 | 1 |
| 14HT | 1-545817 | bg | 2015 | 1 | 0 | 1 | 0 | 1 | 1 | 1 | 1 |
| 14SD | za35988 | cz | 2016 | 1 | 1 | 1 | 1 | 1 | 1 | 1 | 1 |
| 14SF | za60716 | cz | 2016 | 1 | 1 | 1 | 1 | 1 | 1 | 1 | 1 |
| 14TC | za48338 | cz | 2016 | 1 | 1 | 1 | 1 | 1 | 1 | 1 | 1 |
| 14TF | za32371 | cz | 2016 | 1 | 0 | 1 | 0 | 1 | 1 | 1 | 1 |
| 14TS | 1-545764 | bg | 2016 | 1 | 1 | 1 | 1 | 0 | 1 | 0 | 1 |
| 14TZ | 1-545971 | bg | 2016 | 1 | 1 | 1 | 1 | 1 | 1 | 1 | 1 |
| 16BR | 1-555140 | bg | 2016 | 1 | 1 | 1 | 1 | 1 | 1 | 1 | 1 |
| 16EA | fs27400 | ru | 2016 | 1 | 0 | 1 | 0 | 0 | 1 | 0 | 1 |
| 16ER | fs27378 | ru | 2016 | 1 | 1 | 1 | 1 | 0 | 1 | 0 | 1 |
| 16ET | fs27417 | ru | 2016 | 1 | 0 | 1 | 0 | 0 | 1 | 0 | 1 |
| 16EY | 1-545791 | bg | 2016 | 1 | 1 | 1 | 1 | 1 | 1 | 1 | 1 |
| 16FB | 1-545785 | bg | 2016 | 1 | 1 | 1 | 1 | 1 | 1 | 1 | 1 |
| 16FI | 1-545777 | bg | 2016 | 1 | 1 | 1 | 1 | 1 | 1 | 1 | 1 |
| 16FT | 1-545683 | bg | 2016 | 1 | 0 | 1 | 0 | 1 | 1 | 1 | 1 |
| 16GA | za59713 | cz | 2016 | 1 | 1 | 1 | 1 | 1 | 1 | 1 | 1 |
| 16HB | 1-545761 | bg | 2016 | 1 | 1 | 1 | 1 | 1 | 1 | 1 | 1 |
| 16HL | 1-545679 | bg | 2016 | 1 | 1 | 1 | 1 | 1 | 1 | 1 | 1 |
| 16HM | 1-555134 | bg | 2016 | 1 | 1 | 1 | 1 | 1 | 1 | 1 | 1 |
| 18HA | ZA76610 | cz | 2017 | 1 | 0 | 1 | 0 | 1 | 1 | 1 | 1 |
| 18HY | ZA76621 | cz | 2017 | 1 | 1 | 1 | 1 | 1 | 1 | 1 | 1 |
| 18IC | ZA48339 | cz | 2017 | 1 | 1 | 1 | 1 | 1 | 1 | 1 | 1 |
| 18IJ | ZA69547 | cz | 2017 | 1 | 1 | 1 | 1 | 1 | 1 | 1 | 1 |
| 18IQ | ZA76620 | cz | 2017 | 1 | 0 | 1 | 0 | 1 | 1 | 1 | 1 |
| 18KA | ZA69517 | cz | 2017 | 1 | 1 | 1 | 1 | 1 | 1 | 1 | 1 |
| 18LD | ZA44432 | cz | 2017 | 1 | 1 | 1 | 1 | 1 | 1 | 1 | 1 |
| 18LX | ZA45656 | cz | 2017 | 1 | 1 | 1 | 1 | 1 | 1 | 1 | 1 |
| 18LZ | ZA62192 | cz | 2017 | 1 | 1 | 1 | 1 | 1 | 1 | 1 | 1 |

Table S2: The variables used in the models (also see Table S3). We list the abbreviations along with a full description, the units/levels and an annotation whether a variable was used as a response variable (RV), an explanatory variable (EV) or a random factor (RF).

| Variable | Description | Unit/Levels | Used as? |
| --- | --- | --- | --- |
| am.start^1^ | departure for autumn migration | day of the year | RV |
| am.end^1^ | arrival from autumn migration | day of the year | RV |
| sm.star^1^ | departure for spring migration | day of the year | RV |
| sm.end^1^ | arrival from spring migration | day of the year | RV |
| dist^1^ | total migration distance | kilometres | RV |
| migdur^1^ | total migration duration | days | RV |
| speed^1^ | total migration speed | kilometers/day | RV |
| durFB^2^ | flight bout duration | hours | RV |
| durSO^2^ | duration of resting period | hours | RV |
| FH^2^ | flight height | meters a.s.l. | RV |
| int^3,4^ | intensity of infection | relative qPCR intensity | EV |
| season | migration season | am = autumn sm = spring | EV |
| numSex | numerical sex of the host | 1 = male  0 = female  0.5 = NA | EV |
| indi | individual geolocator id | 40 different IDs | RF |
|  | | | |
| ^1^ Unless stated otherwise, we centred this variable within study site by subtracting the site-specific mean from every individual value (indicated by the suffix ‘.pc’). | | | |
| ^2^ For this variable we calculated the mean, median, max and/or sum per individuum to be used in the models. | | | |
| ^3^ This variable was used as unitless log-transformed values. | | | |
| ^4^ We used ‘int1’ when we involved the intensity from the sample before to the tracked migration, ‘int2’ for the sample after or ‘int’ when we used either of the two samples. | | | |
|  | | | |

Table S3: List of model formulas and parameter estimates from models with (A) parasite genus as the only focal explanatory variable, (B) parasite genus and infection intensity as two focal explanatory variables and (C) infection intensity as the only focal explanatory variable (from left to right), and the models with (1) basic migration parameters, (2) migration timing parameters, (3) flight times, (4) resting times and (5) flight heights as response variables (from top to the bottom). Significant estimates and the according variables are shown in bold. The suffix ‘.pc’ indicates that a variable has been centred within each site by subtracting the site-specific mean from the individual values. See Table S2 for a glossary of the variables used in the models.

| (A) GENUS ONLY | (B) LOGST(INT) + GENUS | (C) LOGST(INT) ONLY |
| --- | --- | --- |
| (1) Basic migration parameters | | |
| dist.pc ~ genus + season + Sex + (1 \| indi)  Fixed effects:  Estimate Std. Error df t value Pr(>\|t\|)  (Intercept) 185.55 183.49 35.76 1.011 0.319  genusP -34.00 201.62 42.64 -0.169 0.867  genusH -103.55 196.82 45.69 -0.526 0.601  genusHP -19.90 253.85 40.91 -0.078 0.938  seasonsm 57.61 133.69 16.42 0.431 0.672  Sex -264.32 187.62 25.04 -1.409 0.171 | **dist.pc** ~ **logstint** + genus + season + Sex + (1 \| indi)  Fixed effects:  Estimate Std. Error df t value Pr(>\|t\|)  (Intercept) -429.20 358.41 31.65 -1.198 0.240  **logstint -161.38 76.82 19.26 -2.101 0.049 ***  genusP 225.21 240.02 35.42 0.938 0.354  genusH 483.03 342.50 31.64 1.410 0.168  genusHP 391.16 375.69 38.04 1.041 0.304  seasonsm 149.67 122.05 12.89 1.226 0.242  Sex -269.40 192.06 25.04 -1.403 0.173 | dist.pc ~ logstint + season + Sex + (1 \| indi)  Fixed effects:  Estimate Std. Error df t value Pr(>\|t\|)  (Intercept) 17.17 158.51 47.27 0.108 0.914  logstint -60.11 39.82 48.90 -1.509 0.138  seasonsm 97.22 114.88 26.37 0.846 0.405  Sex -240.66 170.52 36.74 -1.411 0.167 |
| migdur.pc ~ genus + season + Sex + (1 \| indi)  Fixed effects:  Estimate Std. Error df t value Pr(>\|t\|)  (Intercept) 2.750 5.628 53.000 0.489 0.627  genusP -7.347 6.065 53.000 -1.212 0.231  genusH -9.089 6.362 53.000 -1.429 0.159  genusHP -11.889 7.947 53.000 -1.496 0.141  seasonsm 2.834 4.819 53.000 0.588 0.559  Sex 1.932 5.347 53.000 0.361 0.719 | migdur.pc ~ logstint + genus + season + Sex + (1 \| indi)  Fixed effects:  Estimate Std. Error df t value Pr(>\|t\|)  (Intercept) -10.4884 13.2000 52.0000 -0.795 0.430  logstint -3.3509 3.0236 52.0000 -1.108 0.273  genusP -1.1984 8.2101 52.0000 -0.146 0.885  genusH 2.8344 12.4921 52.0000 0.227 0.821  genusHP -0.1707 13.2168 52.0000 -0.013 0.990  seasonsm 3.7986 4.8869 52.0000 0.777 0.440  Sex 2.0243 5.3358 52.0000 0.379 0.706 | **migdur.pc** ~ **logstint** + season + Sex + (1 \| indi)  Fixed effects:  Estimate Std. Error df t value Pr(>\|t\|)  (Intercept) -9.242 4.829 65.000 -1.914 0.0601 .  **logstint -2.768 1.337 65.000 -2.070 0.0424 ***  seasonsm 1.744 4.251 65.000 0.410 0.6829  Sex 4.446 4.532 65.000 0.981 0.3302 |
| speed.pc ~ genus + season + Sex + (1 \| indi)  Fixed effects:  Estimate Std. Error df t value Pr(>\|t\|)  (Intercept) -20.000 30.524 37.480 -0.655 0.5163  genusP 65.833 33.615 43.058 1.958 0.0567 .  genusH 28.576 32.918 45.929 0.868 0.3899  genusHP 70.066 42.288 40.695 1.657 0.1052  seasonsm -9.820 22.614 19.435 -0.434 0.6689  Sex -3.886 31.103 27.587 -0.125 0.9015 | speed.pc ~ logstint + genus + season + Sex + (1 \| indi)  Fixed effects:  Estimate Std. Error df t value Pr(>\|t\|)  (Intercept) 58.9324 63.1840 37.0601 0.933 0.357  logstint 19.8273 13.8971 26.7744 1.427 0.165  genusP 29.8258 41.9806 40.4443 0.710 0.481  genusH -43.6364 60.2706 38.4840 -0.724 0.473  genusHP 0.6138 65.1513 44.0144 0.009 0.993  seasonsm -17.4979 22.6579 17.2428 -0.772 0.450  Sex -5.0208 31.0972 26.5386 -0.161 0.873 | speed.pc ~ logstint + season + Sex + (1 \| indi)  Fixed effects:  Estimate Std. Error df t value Pr(>\|t\|)  (Intercept) 28.398 24.590 55.000 1.155 0.2531  logstint 12.870 6.855 55.000 1.878 0.0658 .  seasonsm -11.601 23.552 55.000 -0.493 0.6243  Sex 1.349 24.877 55.000 0.054 0.9569 |
| (2) Migration timing | | |
| **am.start.pc** ~ **genus1** + numSex  Coefficients:  Estimate Std. Error t value Pr(>\|t\|)  (Intercept) -2.700 4.390 -0.615 0.54414  **genus1P 16.773 5.475 3.064 0.00518 ****  genus1H 10.088 5.507 1.832 0.07889 .  **genus1HP 19.271 7.463 2.582 0.01606 ***  numSex -7.699 4.544 -1.694 0.10268 | am.start.pc ~ logstint1 + genus1 + numSex  Coefficients:  Estimate Std. Error t value Pr(>\|t\|)  (Intercept) 4.093 12.250 0.334 0.7412  logstint1 1.719 2.888 0.595 0.5573  genus1P 13.821 7.441 1.857 0.0756 .  genus1H 4.447 11.000 0.404 0.6896  genus1HP 13.437 12.380 1.085 0.2885  numSex -8.042 4.640 -1.733 0.0959 . | **am.start.pc** ~ **logstint1** + numSex  Coefficients:  Estimate Std. Error t value Pr(>\|t\|)  (Intercept) 13.574 4.308 3.151 0.00339 **  **logstint1 3.638 1.271 2.863 0.00713 ****  numSex -8.040 4.041 -1.990 0.05471 . |
| am.end.pc ~ genus1 + numSex  Coefficients:  Estimate Std. Error t value Pr(>\|t\|)  (Intercept) -2.0701 4.3200 -0.479 0.636  genus1P 3.3621 5.3878 0.624 0.538  genus1H 4.0383 5.4190 0.745 0.463  genus1HP 3.8818 7.3441 0.529 0.602  numSex -0.4723 4.4721 -0.106 0.917 | am.end.pc ~ logstint1 + genus1 + numSex  Coefficients:  Estimate Std. Error t value Pr(>\|t\|)  (Intercept) -4.9882 12.1262 -0.411 0.684  logstint1 -0.7384 2.8590 -0.258 0.798  genus1P 4.6302 7.3663 0.629 0.536  genus1H 6.4619 10.8890 0.593 0.558  genus1HP 6.3881 12.2556 0.521 0.607  numSex -0.3248 4.5936 -0.071 0.944 | am.end.pc ~ logstint1 + numSex  Coefficients:  Estimate Std. Error t value Pr(>\|t\|)  (Intercept) 2.4280 3.9642 0.612 0.544  logstint1 0.9252 1.1691 0.791 0.434  numSex -0.4663 3.7178 -0.125 0.901 |
| sm.start.pc ~ genus2 + numSex  Coefficients:  Estimate Std. Error t value Pr(>\|t\|)  (Intercept) -0.141603 8.245046 -0.017 0.986  genus2P -7.486973 7.699201 -0.972 0.341  genus2H 8.249150 8.548787 0.965 0.344  genus2HP 0.058969 9.687533 0.006 0.995  numSex -0.001944 7.463227 0.000 1.000 | sm.start.pc ~ logstint2 + genus2 + numSex  Coefficients:  Estimate Std. Error t value Pr(>\|t\|)  (Intercept) 1.5097 16.5168 0.091 0.928  logstint2 0.4460 3.8374 0.116 0.908  genus2P -8.3990 11.1090 -0.756 0.457  genus2H 6.5409 17.0961 0.383 0.706  genus2HP -1.5728 17.1764 -0.092 0.928  numSex 0.0631 7.6420 0.008 0.993 | sm.start.pc ~ logstint2 + numSex  Coefficients:  Estimate Std. Error t value Pr(>\|t\|)  (Intercept) 7.4491 5.4190 1.375 0.180  logstint2 0.8066 1.8205 0.443 0.661  numSex -9.1303 6.5896 -1.386 0.176 |
| sm.end.pc ~ genus2 + numSex  Coefficients:  Estimate Std. Error t value Pr(>\|t\|)  (Intercept) 11.809 6.550 1.803 0.084 .  genus2P -7.840 6.117 -1.282 0.212  genus2H -6.382 6.791 -0.940 0.357  genus2HP -8.740 7.696 -1.136 0.267  numSex -8.587 5.929 -1.448 0.160 | sm.end.pc ~ logstint2 + genus2 + numSex  Coefficients:  Estimate Std. Error t value Pr(>\|t\|)  (Intercept) -5.355 12.449 -0.430 0.671  logstint2 -4.635 2.892 -1.603 0.123  genus2P 1.640 8.373 0.196 0.846  genus2H 11.374 12.885 0.883 0.387  genus2HP 8.221 12.946 0.635 0.532  numSex -9.263 5.760 -1.608 0.121 | sm.end.pc ~ logstint2 + numSex  Coefficients:  Estimate Std. Error t value Pr(>\|t\|)  (Intercept) 2.075 3.782 0.549 0.5875  logstint2 -2.307 1.271 -1.816 0.0797 .  numSex -8.685 4.599 -1.888 0.0690 . |
| (3) Flight times | | |
| durFB.mean.pc ~ genus + season + Sex + (1 \| indi)  Fixed effects:  Estimate Std. Error df t value Pr(>\|t\|)  (Intercept) 0.02460 0.37246 41.24017 0.066 0.948  genusP 0.18642 0.39433 47.90401 0.473 0.639  genusH 0.19681 0.41069 50.58490 0.479 0.634  genusHP 0.08910 0.52795 44.05214 0.169 0.867  seasonsm -0.01743 0.27265 30.32173 -0.064 0.949  Sex -0.24174 0.36617 31.83505 -0.660 0.514 | **durFB.mean.pc** ~ **logstint** + genus + season + Sex + (1 \| indi)  Fixed effects:  Estimate Std. Error df t value Pr(>\|t\|)  (Intercept) 1.8224 0.7821 51.6456 2.330 0.0237 *  **logstint 0.4560 0.1768 47.7811 2.579 0.0130 ***  genusP -0.6323 0.4910 51.8964 -1.288 0.2036  genusH -1.4318 0.7440 51.9922 -1.924 0.0598 .  genusHP -1.5806 0.7969 50.6513 -1.983 0.0528 .  seasonsm -0.1375 0.2739 29.9640 -0.502 0.6193  Sex -0.2527 0.3366 29.9887 -0.751 0.4587 | durFB.mean.pc ~ logstint + season + Sex + (1 \| indi)  Fixed effects:  Estimate Std. Error df t value Pr(>\|t\|)  (Intercept) 0.57640 0.35342 53.21645 1.631 0.109  logstint 0.13786 0.09454 64.87535 1.458 0.150  seasonsm -0.10354 0.26659 36.09857 -0.388 0.700  Sex -0.39837 0.34961 35.73147 -1.139 0.262 |
| durFB.max.pc ~ genus + season + Sex + (1 \| indi)  Fixed effects:  Estimate Std. Error df t value Pr(>\|t\|)  (Intercept) -1.9448 1.7410 32.3423 -1.117 0.272  genusP 0.1341 1.8420 42.9742 0.073 0.942  genusH 0.9023 1.9177 48.1383 0.471 0.640  genusHP -1.6268 2.4671 36.7901 -0.659 0.514  seasonsm 0.6623 1.2685 19.7072 0.522 0.607  Sex 1.6716 1.7133 21.3003 0.976 0.340 | durFB.max.pc ~ logstint + genus + season + Sex + (1 \| indi)  Fixed effects:  Estimate Std. Error df t value Pr(>\|t\|)  (Intercept) 1.3160 3.8155 49.8441 0.345 0.732  logstint 0.8203 0.8535 39.6182 0.961 0.342  genusP -1.3539 2.4088 51.8865 -0.562 0.577  genusH -2.0712 3.6407 51.2685 -0.569 0.572  genusHP -4.5628 3.9306 51.2861 -1.161 0.251  seasonsm 0.4340 1.2904 19.9101 0.336 0.740  Sex 1.6329 1.7162 21.7179 0.951 0.352 | durFB.max.pc ~ logstint + season + Sex + (1 \| indi)  Fixed effects:  Estimate Std. Error df t value Pr(>\|t\|)  (Intercept) -0.2280 1.6748 52.8694 -0.136 0.892  logstint 0.2991 0.4233 63.1852 0.707 0.482  seasonsm -0.3032 1.0928 35.6097 -0.277 0.783  Sex 1.4059 1.7303 37.0539 0.813 0.422 |
| (4) Resting times | | |
| durSO.mean.pc ~ genus + **season** + Sex + (1 \| indi)  Fixed effects:  Estimate Std. Error df t value Pr(>\|t\|)  (Intercept) -1.1759 1.0067 38.3077 -1.168 0.2500  genusP 0.8055 1.0702 45.6124 0.753 0.4555  genusH -0.1134 1.1170 48.6977 -0.102 0.9196  genusHP -1.1809 1.4288 39.8855 -0.827 0.4134  seasonsm 2.0587 0.7587 27.0921 2.713 0.0114 *  Sex -1.3849 0.9839 27.9010 -1.408 0.1703 | durSO.mean.pc ~ logstint + genus + **season** + Sex + (1 \| indi)  Fixed effects:  Estimate Std. Error df t value Pr(>\|t\|)  (Intercept) -2.64016 2.24882 51.08715 -1.174 0.24583  logstint -0.36713 0.50526 45.14071 -0.727 0.47121  genusP 1.48508 1.41670 51.99960 1.048 0.29937  genusH 1.21676 2.14326 51.80493 0.568 0.57268  genusHP 0.08963 2.30678 51.19049 0.039 0.96916  seasonsm 2.15952 0.77110 26.99632 2.801 0.00931 **  Sex -1.35880 0.99371 28.14858 -1.367 0.18232 | durSO.mean.pc ~ logstint + **season** + Sex + (1 \| indi)  Fixed effects:  Estimate Std. Error df t value Pr(>\|t\|)  (Intercept) -1.4658 0.8462 52.6913 -1.732 0.0891 .  logstint -0.1456 0.2325 61.0186 -0.626 0.5335  seasonsm 1.9585 0.7202 35.7824 2.719 0.0100 *  Sex -1.0992 0.8075 33.8499 -1.361 0.1825 |
| durSO.max.pc ~ **genus** + season + Sex + (1 \| indi)  Fixed effects:  Estimate Std. Error df t value Pr(>\|t\|)  (Intercept) 22.67 58.24 39.56 0.389 0.69918  genusP -118.92 61.45 47.55 -1.935 0.05893 .  genusH -116.58 63.88 50.82 -1.825 0.07391 .  **genusHP -268.92 82.42 43.91 -3.263 0.00214 ****  seasonsm 34.31 41.70 27.50 0.823 0.41773  Sex 70.33 57.51 29.60 1.223 0.23100 | durSO.max.pc ~ logstint + genus + season + Sex + (1 \| indi)  Fixed effects:  Estimate Std. Error df t value Pr(>\|t\|)  (Intercept) -54.24 127.07 50.41 -0.427 0.671  logstint -19.28 28.32 43.08 -0.681 0.500  genusP -84.16 80.35 51.80 -1.047 0.300  genusH -45.95 121.36 51.33 -0.379 0.707  genusHP -199.84 131.34 51.81 -1.521 0.134  seasonsm 39.42 42.50 26.70 0.927 0.362  Sex 71.67 58.00 29.15 1.236 0.226 | durSO.max.pc ~ **logstint** + season + Sex + (1 \| indi)  Fixed effects:  Estimate Std. Error df t value Pr(>\|t\|)  (Intercept) -131.79 49.14 52.97 -2.682 0.00974 **  **logstint -39.24 13.38 63.57 -2.932 0.00467 ****  seasonsm 23.23 39.58 35.68 0.587 0.56089  Sex 63.98 47.57 34.40 1.345 0.18748 |
| (5) Flight height | | |
| FH.mean.pc ~ genus + season + Sex + (1 \| indi)  Fixed effects:  Estimate Std. Error df t value Pr(>\|t\|)  (Intercept) -107.18 103.63 42.36 -1.034 0.307  genusP 128.20 106.30 51.43 1.206 0.233  genusH 190.87 108.74 52.99 1.755 0.085 .  genusHP 126.45 143.27 51.61 0.883 0.382  seasonsm -2.10 64.00 27.89 -0.033 0.974  Sex 26.08 105.28 33.16 0.248 0.806 | FH.mean.pc ~ logstint + genus + season + Sex + (1 \| indi)  Fixed effects:  Estimate Std. Error df t value Pr(>\|t\|)  (Intercept) 228.77 206.99 49.28 1.105 0.2744  logstint 82.46 45.42 40.13 1.816 0.0769 .  genusP -32.43 131.59 50.86 -0.246 0.8063  genusH -119.88 198.24 50.14 -0.605 0.5481  genusHP -183.91 216.41 51.92 -0.850 0.3993  seasonsm -24.89 66.48 26.43 -0.374 0.7111  Sex 20.67 99.91 30.85 0.207 0.8375 | FH.mean.pc ~ **logstint** + season + Sex + (1 \| indi)  Fixed effects:  Estimate Std. Error df t value Pr(>\|t\|)  (Intercept) 104.16 84.57 51.44 1.232 0.22370  **logstint 56.93 21.16 62.21 2.691 0.00915 ****  seasonsm -25.94 54.03 33.43 -0.480 0.63427  Sex 40.98 87.88 35.13 0.466 0.64392 |
| FH.max.pc ~ genus + season + Sex + (1 \| indi)  Fixed effects:  Estimate Std. Error df t value Pr(>\|t\|)  (Intercept) 94.94 297.38 36.03 0.319 0.751  genusP -544.89 312.65 46.40 -1.743 0.088 .  genusH -193.21 324.40 50.84 -0.596 0.554  genusHP -273.62 419.99 42.68 -0.651 0.518  seasonsm 204.82 208.28 22.67 0.983 0.336  Sex 47.29 294.93 25.29 0.160 0.874 | FH.max.pc ~ logstint + **genus** + season + Sex + (1 \| indi)  Fixed effects:  Estimate Std. Error df t value Pr(>\|t\|)  (Intercept) 1115.66 639.66 51.78 1.744 0.0871 .  logstint 236.06 145.30 46.72 1.625 0.1110  **genusP -1142.93 400.39 51.31 -2.855 0.0062 ****  genusH -1117.70 607.47 51.94 -1.840 0.0715 .  genusHP -1250.35 648.05 48.27 -1.929 0.0596 .  seasonsm 117.63 228.09 22.43 0.516 0.6111  Sex 42.39 269.50 21.52 0.157 0.8765 | FH.max.pc ~ logstint + season + Sex + (1 \| indi)  Fixed effects:  Estimate Std. Error df t value Pr(>\|t\|)  (Intercept) -20.10 279.78 51.00 -0.072 0.943  logstint 13.01 70.60 62.73 0.184 0.854  seasonsm 42.11 181.96 32.71 0.231 0.818  Sex 75.81 289.30 34.21 0.262 0.795 |


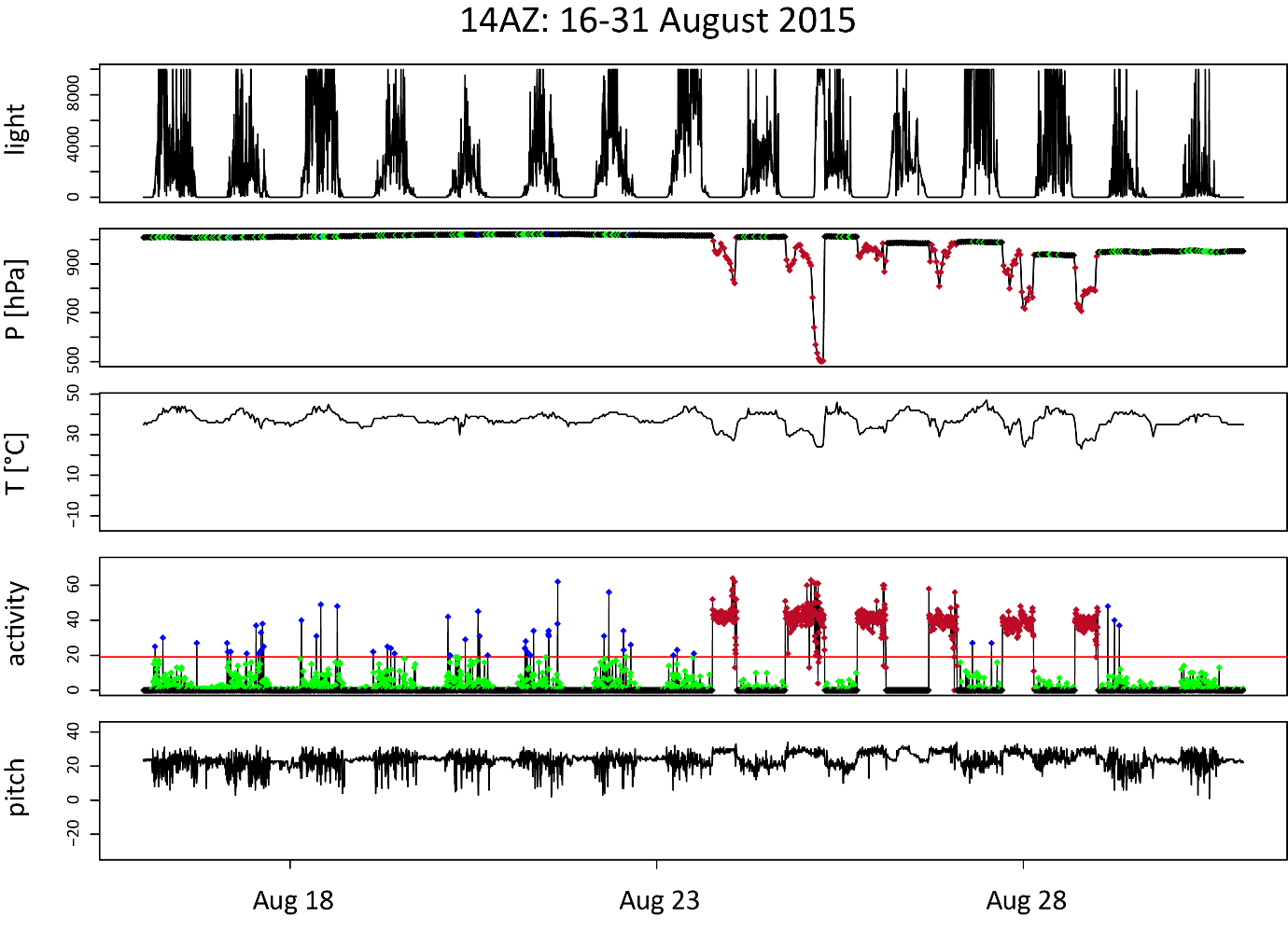


Figure S1: A sample data set for the period 16-31 August 2015 from a great reed warbler with the multi-sensor logger ID 14AZ. The panels depict data from the different sensors (from top to the bottom): light intensity, air pressure, ambient temperature, locomotor activity and pitch (i.e. mean body position). The colours in the pressure and activity panels show behaviours categorised as ‘resting’ (black), ‘active’ (green), ‘flight’ (blue) and ‘migratory flight’ (red).
